# Multimodal MRI of myalgic encephalomyelitis/chronic fatigue syndrome: A cross-sectional neuroimaging study toward its neuropathophysiology and diagnosis

**DOI:** 10.3389/fneur.2022.954142

**Published:** 2022-09-16

**Authors:** Zack Y. Shan, Abdalla Z. Mohamed, Thu Andersen, Shae Rendall, Richard A. Kwiatek, Peter Del Fante, Vince D. Calhoun, Sandeep Bhuta, Jim Lagopoulos

**Affiliations:** ^1^Thompson Institute, University of the Sunshine Coast, Birtinya, QLD, Australia; ^2^Tri-institutional Center for Translational Research in Neuroimaging and Data Science (TReNDS), Georgia State University, Georgia Institute of Technology, Emory University, Atlanta, GA, United States; ^3^Medical Imaging Department, Gold Coast University Hospital, Parklands, QLD, Australia

**Keywords:** translational neuroimaging, MRI, ME/CFS, neuromarker, neurovascular coupling

## Abstract

**Introduction:**

Myalgic encephalomyelitis/chronic fatigue syndrome (ME/CFS), is a debilitating illness affecting up to 24 million people worldwide but concerningly there is no known mechanism for ME/CFS and no objective test for diagnosis. A series of our neuroimaging findings in ME/CFS, including functional MRI (fMRI) signal characteristics and structural changes in brain regions particularly sensitive to hypoxia, has informed the hypothesis that abnormal neurovascular coupling (NVC) may be the neurobiological origin of ME/CFS. NVC is a critical process for normal brain function, in which glutamate from an active neuron stimulates Ca^2+^ influx in adjacent neurons and astrocytes. In turn, increased Ca^2+^ concentrations in both astrocytes and neurons trigger the synthesis of vascular dilator factors to increase local blood flow assuring activated neurons are supplied with their energy needs.

This study investigates NVC using multimodal MRIs: (1) hemodynamic response function (HRF) that represents regional brain blood flow changes in response to neural activities and will be modeled from a cognitive task fMRI; (2) respiration response function (RRF) represents autoregulation of regional blood flow due to carbon dioxide and will be modeled from breath-holding fMRI; (3) neural activity associated glutamate changes will be modeled from a cognitive task functional magnetic resonance spectroscopy. We also aim to develop a neuromarker for ME/CFS diagnosis by integrating the multimodal MRIs with a deep machine learning framework.

**Methods and analysis:**

This cross-sectional study will recruit 288 participants (91 ME/CFS, 61 individuals with chronic fatigue, 91 healthy controls with sedentary lifestyles, 45 fibromyalgia). The ME/CFS will be diagnosed by consensus diagnosis made by two clinicians using the Canadian Consensus Criteria 2003. Symptoms, vital signs, and activity measures will be collected alongside multimodal MRI.

The HRF, RRF, and glutamate changes will be compared among four groups using one-way analysis of covariance (ANCOVA). Equivalent non-parametric methods will be used for measures that do not exhibit a normal distribution. The activity measure, body mass index, sex, age, depression, and anxiety will be included as covariates for all statistical analyses with the false discovery rate used to correct for multiple comparisons.

The data will be randomly divided into a training (*N* = 188) and a validation (*N* = 100) group. Each MRI measure will be entered as input for a least absolute shrinkage and selection operator—regularized principal components regression to generate a brain pattern of distributed clusters that predict disease severity. The identified brain pattern will be integrated using multimodal deep Boltzmann machines as a neuromarker for predicting ME/CFS fatigue conditions. The receiver operating characteristic curve of the identified neuromarker will be determined using data from the validation group.

**Ethics and study registry:**

This study was reviewed and approved by University of the Sunshine Coast University Ethics committee (A191288) and has been registered with The Australian New Zealand Clinical Trials Registry (ACTRN12622001095752).

**Dissemination of results:**

The results will be disseminated through peer reviewed scientific manuscripts and conferences and to patients through social media and active engagement with ME/CFS associations.

## Introduction

Myalgic encephalomyelitis/chronic fatigue syndrome (ME/CFS) is a serious, long-term illness for which the underlying disease process remains unknown, and no objective diagnostic test exists. The disease is characterized by profound fatigue for more than 6 months, cognitive and motor dysfunction, unrefreshing sleep, and/or orthostatic intolerance (1). There are 17 to 24 million people affected by ME/CFS, with 25% of them housebound (2). In addition to enduring the disease itself and its economic burden, 80% of ME/CFS patients struggle to get a diagnosis and are often left depressed by the lack of a diagnostic certainty and medical understanding of the disease processes. Although the etiology of ME/CFS remains unresolved, the well-documented autonomic dysfunction, sleep disturbance, cognitive impairments, altered sensory and pain perception, and reduced motor speed suggest that abnormal brain function plays a crucial role in the underlying disease process of ME/CFS (3). As such, ME/CFS has been classified as a neurological disease (ICD code 10 G93.3) by the WHO.

Additional brain area recruitment associated with cognitive tasks during task fMRI is one of the most frequently observed differences between patients with ME/CFS and controls (4). The task fMRI signal was based on neurovascular coupling (NVC), the dynamic regulation of blood flow induced by neural activity. NVC is a critical process for normal brain function, in which glutamate from an active neuron stimulates Ca^2+^ influx in adjacent neurons and astrocytes. In turn, increased Ca^2+^ concentrations in both astrocytes and neurons trigger the synthesis of vascular dilation factors to increase local blood flow, ensuring activated neurons are supplied with their energy needs (5, 6). Previous genetic and electrophysiological studies showed that transient receptor potential melastatin subfamily 3 (TRPM3) activity and Ca^2+^ mobilization were reduced in ME/CFS (7–9). Reduced Ca^2+^ mobilization may delay NVC and result in the less responsive fMRI signals (i.e., coupled less tightly with tasks with lower sample entropies) that were observed in our previous neuroimaging study (10). The delayed NVC ensues subtle brain changes, culminating in chronic brain injury and associated cognitive impairment (5), presenting as structural changes in brain regions (11–16) susceptible to hypoxia. Moreover, we postulate that a higher level of glutamate is released because of delayed NVC in ME/CFS patients and results in additional brain area recruitment during tasks (4, 10). Excess glutamate may cause excitotoxicity, which is consistent with increased expression of the vasoactive intestinal peptide receptor 2 observed in ME/CFS (17). The intestinal peptide receptor 2 is a neuroprotective agent against excitotoxicity and is released in response to high levels of glutamate (18). Thus, we hypothesize that delayed NVC may explain hypoxia-related brain structural changes and excess glutamate in ME/CFS, which further impairs the brain because of excitotoxicity.

Our previous studies have described brain differences in ME/CFS, related to the brain structure, functional networks (11–13, 15, 16, 19), and BOLD signal changes (10, 20). Although none of these differences alone can differentiate ME/CFS from normal and other disorders, they suggested that a unique brain signature for ME/CFS may be identifiable. Thus, we will also use the least absolute shrinkage and selection operator—regularized principal components regression (LASSO-PCR) (21), to identify brain signatures that contribute to reliable prediction. Then multimodal deep Boltzmann machine (DBM) (22) will be used to integrate multimodal signatures with cross-modality information to develop a neuromarker for objective diagnosis of ME/CFS.

The present paper summarizes the research protocol that addresses the aforementioned aims. We report the analysis plans here to reduce the file-drawer effect and improve the study reproducibility, which is currently solicited in translational research (23) and more urgently required in ME/CFS because of the controversy associated with this ill-defined disease (24).

## Methods and analysis

### The study design

**Figure 1:**
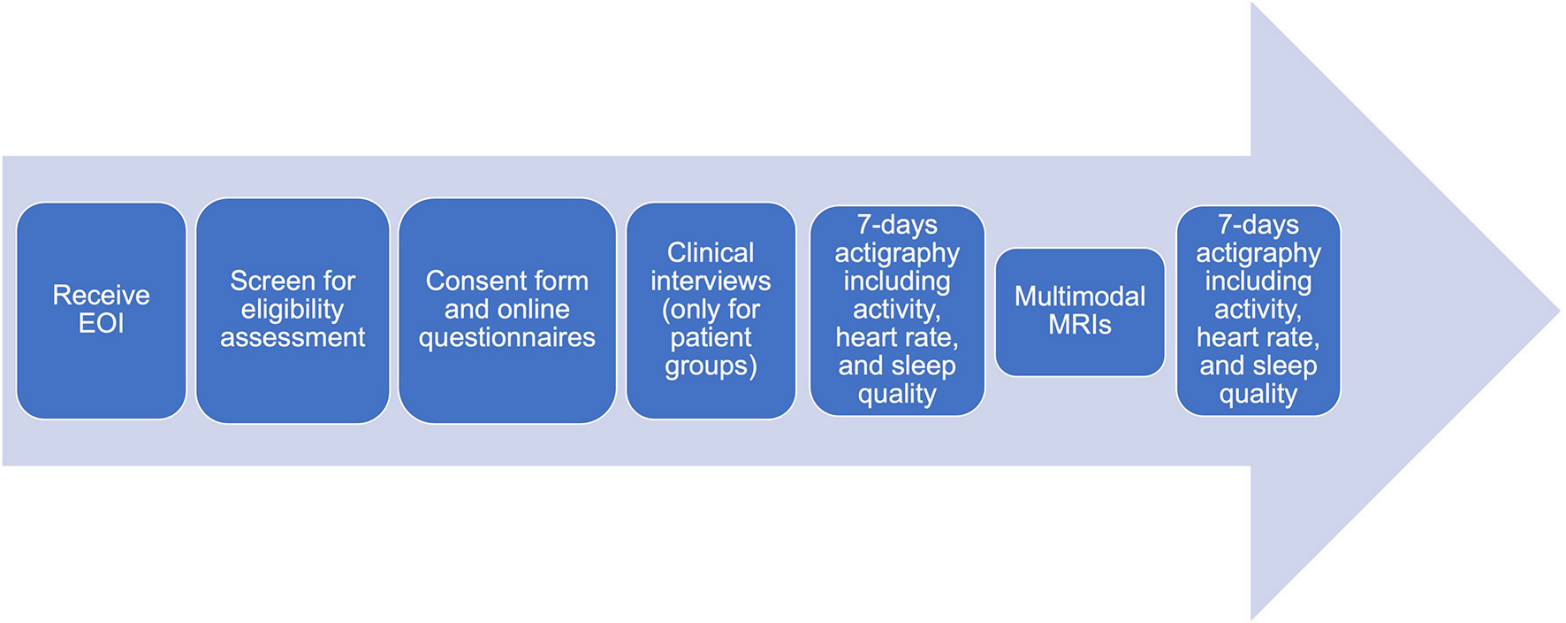
Flowchart for data collection.

The multimodal MRIs of ME/CFS is a cross-sectional study that commenced in January 2020 and will be completed in December 2024. Participants who express an interest will be screened for eligibility according to inclusion/exclusion criteria. Symptom scores, activity levels, MRIs (detailed below) will be collected after receiving written consent from eligible participants (Figure 1).

### The primary outcomes

#### Aim 1

To establish *group* differences in Hemodynamic Response Function (HRF) and its relationship to fatigue severity in ME/CFS patients using functional MRI (fMRI).

#### Aim 2

To establish *group* differences in dynamic glutamate activity during cognitive tasks and its relationship to fatigue severity in ME/CFS patients using functional magnetic resonance spectroscopy (fMRS).

#### Aim 3

To develop a neuromarker for ME/CFS diagnosis. The neuromarker will be relevant brain features integrated from multiple MRI modalities using a deep learning framework and validated using independent data.

### The secondary outcomes

i To compare brain structural and functional differences and associations with symptoms among ME/CFS, fibromyalgia, chronic fatigue, and healthy controls (HCs).
ii To establish *group* differences in high-frequency heart rate variability and associated fMRI signal changes in ME/CFS.
iii To establish *group* objective sleep quality differences and associated brain structure and function differences in ME/CFS.
iv To establish *group* differences in sleep measures and changes low- and high-frequency heart rate variability during awake and sleep before and after MRI scanning.
v To establish the repeatability of fMRS measures.

### The recruitment and selection of the sample population

We will recruit 288 adult participants (18–65 years old), including 91 patients with ME/CFS, 61 individuals with chronic fatigue, 91 HCs with sedentary lifestyles, and 45 patients with fibromyalgia. The risk of an ill-defined ME/CFS patient cohort will be mitigated by employing a consensus diagnosis of ME/CFS from two clinicians (R.A.K and P.D., both have over 10 years of experience in ME/CFS) and using the Canadian Consensus Criteria (CCC) 2003 (1). This study will also include groups of fibromyalgia, a frequent co-morbidity of ME/CFS. The American College of Rheumatology (ACR) 2016 fibromyalgia criteria (25) will be used for screening and followed by clinic diagnosis by a rheumatologist (R.A.K.). The chronic fatigue is defined as self-identified ME/CFS but not confirmed by the two clinicians (i.e., present with other conditions or lack core ME/CFS symptoms) or with other disorders (not ME/CFS or fibromyalgia) that fatigue is a significant symptom. These two groups are included to ensure specificity of the identified neuromarker. Patients with ME/CFS have reduced daily activity because of their illness. However, a sedentary lifestyle itself may affect brain structure and function. Therefore, there is a risk of identifying brain structural or functional differences from ME/CFS sequelae, rather than from the illness per se. This study will mitigate against this problem by selecting HCs with sedentary lifestyles. A sedentary lifestyle will be defined as spending <60 min in moderate or high-intensity activity (i.e., exercise) per week (26). Furthermore, activity level will be monitored by the Actigraph GT3X-BT device (ActiGraph LLC., United States) for 14 days. The recorded activity level will be included as covariates for all statistical analyses but will not change participants' eligibility.

The inclusion criteria include adult individuals who express an interest, meet each group definition, and are confirmed by clinicians.

The exclusion criteria include individuals (1) out of the age range, (2) pregnant, (3) with severe intellectual or mental impairment preventing them from fully understanding the study to give consent, (4) with mental disorders including post-traumatic stress disorder, obsessive compulsive disorder, schizophrenia, and bipolar disorder, (5) with a known other neurological disorder, (6) who cannot read and communicate in English, (7) recruited by supervising relationship or where a conflict of interest exists, (8) with alcohol or substance related disorder, (9) with BMI >35, (10) smoking (including marijuana or substance usage), (11) with diabetes, hypertension, or uncontrolled hyperlipidaemia, (12) currently on medication acting on the brain, (13) with a clinical diagnosis of hemochromatosis, (14) with a double copy of the Haemochromatosis gene, (15) who experienced migraines more than six times a year before the onset of their symptoms. A researcher will check the exclusion criteria at the screening stage. After the screening process, a differential diagnostic list will be completed by each participant and discussed with two clinicians for diagnosis of ME/CFS during clinical interviews.

Additional exclusion criteria for HCs include individuals (1) with a chronic disease, i.e., a condition that last 1 year or more and require ongoing medical attention or limit activities of daily living or both, including heart disease, stroke, lung cancer, colorectal cancer, depression, type 2 diabetes, arthritis, osteoporosis, asthma, chronic obstructive pulmonary disease, chronic kidney disease, and (2) who experience migraines more than six times a year, and (3) who do more than 60 min in moderate or high-intensity activity per week.

### Data collections

#### Symptom questionnaires

After informed consent has been obtained, the following assessments will be undertaken: (1) symptom information relevant to establishing CCC ME/CFS classification (1); (2) the 36-item Short-Form Health Survey; (c) Hospital Anxiety and Depression Scale questionnaire; (d) the Bell disability score; (e) The Assessment of Quality of Life questionnaire; (g) the Pittsburgh sleep quality index questionnaire.

#### Clinical interviews

Two clinicians (R.A.K and P.D.) will perform a remote interview independently with patients. Prior to the clinical interviews, height, weight, BMI, blood pressure, pulse rate, oxygen saturation, weighted standing time for evaluation of postural orthostatic tachycardia syndrome (POTS) (27), and the Beighton scores (a measure of generalized joint hypermobility) (28) for each participant will be collected and shared with the clinician. The clinician requested the POTS and the Beighton scores to assist diagnosis of ME/CFS. None of the POTS or the Beighton scores alone differentiates ME/CFS but will be used with other measures by clinicians.

#### Actigraphy

Participants will be given a GT3X-BT device with a heart rate monitor to wear 7 days before the MRI. The heart rate monitor is to be worn for 24 h. After the MRI scan, participants will be given another GT3X-BT device to wear for 7 days with a 24-h heart rate monitor to be worn on the next morning of the MRI scan. We will collect actigraphy data before and after the MRI scanning (i) to obtain a robust estimations of activity levels, sleep qualities, and heart rate variabilities and (ii) to investigate sleep quality and heart rate variability changes after a moderate exertion (MRI scanning) in ME/CFS.

#### Multimodal MRI

**Figure 2:**
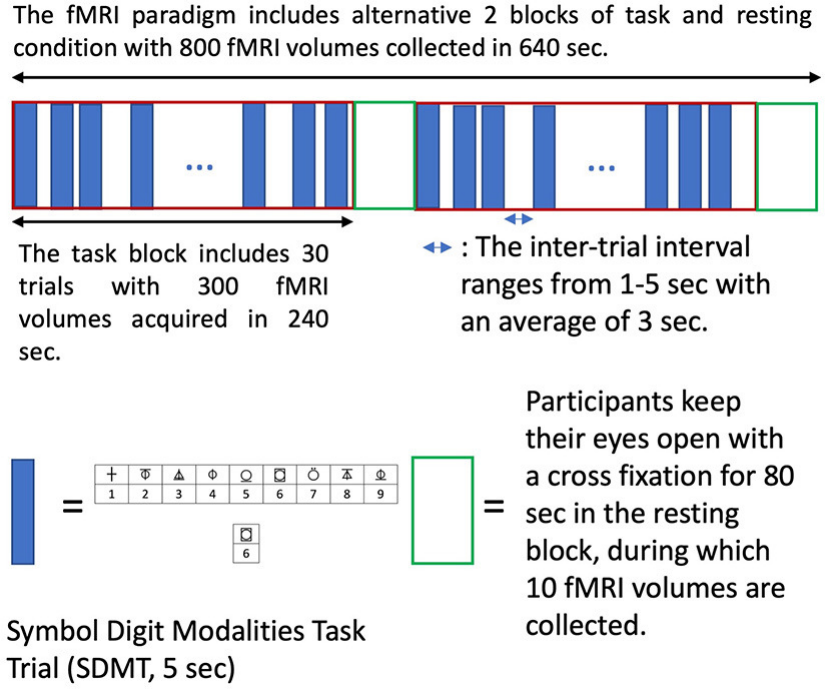
The semi-random design of task fMRI paradigm. The semi-random task paradigm (maximizing both estimation efficiency and detection power) includes two alternative tasks and resting conditions blocks (640 sec). Each task block has 30 symbol digit modalities task (SDMT) trials. Each trial takes 5s with a random inter-trial interval ranging from 1–5s (average of 3s). The SMDT requires participants to determine if the lower symbol digit pair agrees with upper symbol-digit references and respond with yes and no keys. Participants keep their eyes open with a cross fixation during the inter-trial intervals and the resting blocks.

**Figure 3:**
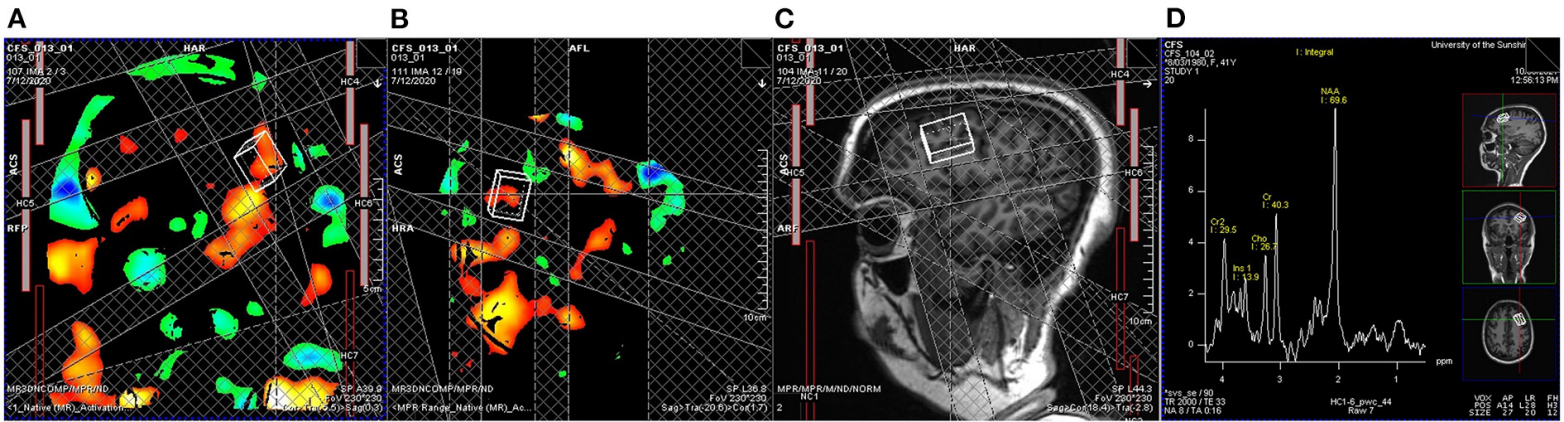
Real-time task fMRI guided functional magnetic resonance spectroscopy (fMRS). A general linear model determined BOLD signal changes associated with the symbol digit modalities test implemented on the MRI scanner with a threshold of uncorrected *P* < 0.001. A cuboid voxel covering the left dorsal prefrontal cortex (20 mm from superior to inferior, 27 mm from anterior to posterior, and 12 mm from left to right) is adjusted according to BOLD activation maps, **(A)** coronal and **(B)** sagittal views, and **(C)** T1-weighted anatomic images to cover left dorsal prefrontal cortex. **(D)** An example of a measurement averaged from 8 volumes from another participant shows the exact anatomical location and the acceptable signal-to-noise ratio for estimating the glutamate level.

Brain images are acquired using a 3T MRI scanner with a 64-channel head coil (Skyra, Siemens) at the Thompson Institute (TI), University of the Sunshine Coast (UniSC). A fatigue state questionnaire that measures current fatigue levels (29) will be completed before and after the MRI for each participant. The MRIs will include:

I Structural MRI (sMRI) using a T1-weighted magnetization prepared rapid gradient-echo sequence (MPRAGE): 208 slices, dimension 256 × 256, voxel size 1 mm × 1 mm × 1 mm, TR/TE 2,200/1.71 ms, flip angle 7°.
II A set of resting-state fMRI (rsfMRI, 192 volumes) using a multiband EPI sequence: 108 slices, dimension 126 × 126, multiband = 4, dimension 138 × 138, 1.6 mm^3^ isotropic voxel, TR/TE 2,500/42 ms, flip angle 75°. Participant will be instructed to keep their eyes open with fixation of a cross for 8 min acquisition. Heart rate will be recorded simultaneously using pulse oximetry from the MRI scanner.
III A set of task fMRI (tfMRI, 800 volumes) using a multiband EPI sequence (60 slices, multiband = 8, dimension 74 × 74, 3 mm^3^ isotropic voxels, TR/TE 800/33 ms, flip angle 65°) while participants perform a symbol digit modalities test (SDMT), which is widely used for cognitive evaluation of information processing speed. A semi-random design, which simultaneously achieves maximum estimation efficiency and detection power, (30) was used (Figure 2). The paradigm is incorporated into the scanner's fMRI sequence for general linear modeling of BOLD signal changes associated with tasks. Brain regions with BOLD signal changes associated tasks are used for voxel location of functional magnetic resonance spectroscopy (fMRS).
IV A set of breath-holding fMRI (BH fMRI, 324 volumes) will be acquired using the same pulse sequence and parameters as tfMRI above. Participants perform 6 BH tasks; each BH task consists of 14.4 s (18 fMRI volumes) natural breath, 3 paced breathing (4.8 s each with 2.4 s breath in and out, respectively) of 14.4 s and a 14.4 s end-expiration breath-hold.
V A set of fMRS using the HERMES (Hadamard Encoding and Reconstruction of MEGA-Edited Spectroscopy) acquisition method with TR/TE = 2,000/23 ms. The task paradigm is the same as the tfMRI paradigm (Figure 2), during which 16 and 5 measurements (averaged from 8 volumes per measurement) are collected for task and resting block, respectively. A single cuboid voxel, 20 mm (superior-inferior), 27 mm (anterior-posterior), and 12 mm (left-right), covering the left dorsal prefrontal cortex, is adjusted according to individual BOLD activation maps (Figure 3).
VI A set of diffusion tensor images (DTI) using a multiband EPI sequence (72 slices, multiband factor 3, dimension = 114 × 114, voxel size 2 × 2 × 2 mm^3^, TR/TE = 4,500/123 ms; free diffusion mode, 96 diffusion directions, bipolar diffusion scheme with two diffusion weighting of b = 0 and b = 2,500, phase encoding direction = anterior to posterior). Six volumes of b = 0 with phase encoding direction in posterior to anterior are also collected for the eddy current correction.
VII A final set of rsfMRI will be collected using the same setting as II.

### Data analysis plan

The measured variables will firstly be tested for normal distribution. Equivalent non-parametric methods will be used if any variable does not exhibit a normal distribution. The activity measure, body mass index, age, depression, and anxiety will be included as covariates for all statistical analyses. The false discovery rate (FDR) will be used to correct for multiple comparisons.

#### Actigraphy data

The metabolic equivalent of the task from the GT3X_BT watch will be averaged over 14 days as the activity measure for each participant. The sleep outputs of the GT3X-BT watch, including latency, efficiency, total time in bed, total sleep time, wake after sleep onset, number of awakenings, and averaged awakening time calculated using the Cole-Kripke algorithm (31), will be averaged as objective sleep measurements for each participant. The 24-h heart rate will be downloaded and averaged to generate low- (< 0.1 Hz) and high-frequency (> 0.2 Hz) during awake and sleep for each participant.

#### Preprocessing of MRIs

sMRI data will be processed using an optimized SPM12 (Statistical Parametric Mapping, Wellcome Trust Center for Neuroimaging, London, United Kingdom) procedure validated in our previous study (15) to generate the gray matter (GM) and white matter (WM) density maps (for Aim3). With the notion that our scanner delivers 5 pulses without image acquisition for signal stabilization, standard preprocessing of fMRI data will be used, including motion, distortion, and slice timing corrections, normalization to standard space, and motion scrubbing and physiological noise removal using the RETROICOR approach (32). Pre-processing of fMRS will include phase-, shift-, and eddy current correction and removal of the first fMRS measurement for each condition. Two consecutive spectra in each condition will be averaged to increase the signal-to-noise ratio. Pre-processing of DTI will include correction of image distortions from eddy currents and detection and replacement of outliers due to the head and cardiac pulsatile motion. The HRF features (for Aim 1 and Aim 3) will be modeled using sHRF toolkit developed in our previous study (33) based on tfMRI. The sample entropy (SampEn) map of BOLD signal changes (for Aim 3) will be calculated as in our previous study (10) based on tfMRI. The fractional amplitude of low-frequency fluctuation (fALFF) map of task residue BOLD signal changes (for Aim 3) will be calculated as the power within the low-frequency range (0.01–0.1Hz) divided by the total power in the entire detectable frequency range (34) after removing task-related activity with the general linear modeling (35). Resting glutamate levels and dynamic glutamate changes in task conditions (for Aim 2) will be calculated using ‘LCModel' (version 6.3) with a simulated basis set for prior knowledge (36). The apparent diffusion coefficient and fractional anisotropy maps (for Aim 3) will be calculated using the FMRIB Software Library (FSL) based on diffusion MRI data. The RRF (for Aim 1 and 3) will be modeled similarly to our previous study (33) with an RRF which is slower than neuronally-induced BOLD signal changes (37) based on the breath-holding tfMRI.

#### Group difference in hemodynamic response function (HRF) (Aim 1)

The HRF features will be compared using one-way analysis of covariance (ANCOVA) among the four groups. The respiration response function (RRF) will be modeled in the breath-holding tfMRI data. This will enable us to disentangle non-neuronally-induced cerebrovascular reactivity from the neurovascular coupling effects. The RRF will be summarized similarly to the HRF by amplitude (*H*), time to peak (*T*), onset (*O*), and width (*W*). The RRF features at the same brain location will be used as a covariate in the HRF ANCOVA analysis to exclude confounding cerebrovascular reactivity (e.g., RRF-*H* as a covariate for HRF-*H* comparison, etc.,). Group differences in each RRF feature will also be tested using ANCOVA among the four groups. The bivariate correlation will be tested between the HRF/RRF features and the health scores. Additionally, the extent to which these correlated features explain variance in the health scores after accounting for all other variables will be evaluated using hierarchical regression.

#### Group difference in dynamic glutamate response induced by cognitive task (Aim 2)

To directly test our hypothesis of an elevated dynamic glutamate response to cognitive tasks, dynamic glutamate response will be compared among the four groups using ANCOVA. In addition, dynamic glutamate response provides another measure of neural activity, which is considerably less sensitive to vascular changes. Establishing these differences will enable us to further elucidate NVC abnormality in ME/CFS. Bivariate correlations will be tested between the dynamic glutamate responses and health scores. In addition, the extent to which these correlated responses explain variance in the health scores after accounting for all other variables will be evaluated using hierarchical regression.

#### Neuromarker identification and validation (Aim 3)

The data will be randomly divided into a model group (168 samples from 51 ME/CFS, 41 fatigue conditions, 51 HCs, and 25 fibromyalgia), as well as a validation group (120 samples from 40 ME/CFS, 20 fatigue conditions, 40 HCs, and 20 fibromyalgia). The region-based HRF features, RRF features, sample entropy, and fractional amplitude of low-frequency fluctuations will be converted into whole-brain voxel-wise maps by assigning regional measures to each voxel within the region and zeros outside. Each MRI measure (GM and WM density map, HRF features, etc.) will be entered as input for least absolute shrinkage and selection operator—regularized principal components regression (LASSO-PCR) (21) to generate a brain pattern of distributed clusters that predict disease severity. The identified brain pattern will be integrated using multimodal deep Boltzmann machines (DBM) (22) as a neuromarker to predict ME/CFS. The importance of each cluster in the brain pattern will be determined by the Kullback-Leibler divergence of the prediction distributions with and without it. Clusters with no significant information gain will be removed. The receiver operating characteristic (ROC) curve of the identified neuromarker will be determined using data from the validation group.

### Power analysis

**Figure 4:**
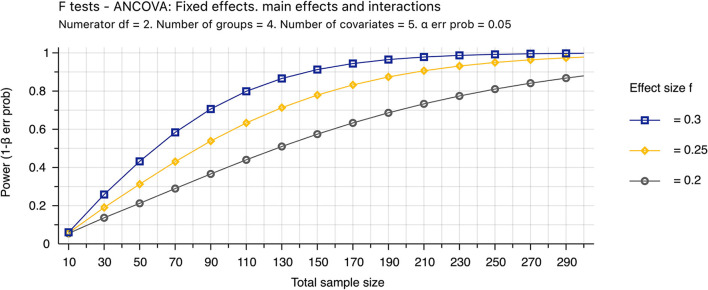
Power analysis. The theoretic sample size was determined by the sensitivity value (effect size = 0.25) that further decreasing the effect size from 0.25 to 0.2 requires appreciably increasing the sample size from 251 to 390. The sample size assumes that 87% of data are valid (*N* = 251/0.87 ~ 288). Invalid data include MRI images with excessive motions and new conditions found after completion of the study, and the percentage was estimated empirically. The analysis of covariance (ANCOVA) will be used to determine the mean difference among four groups with 5 covariates of age, gender, activity levels, body mass index, depression, and anxiety. We assumed both type I and II errors of 0.05.

The HRF features and dynamic glutamate changes associated with a cognitive task in individuals with fatigue and ME/CFS will be tested for the first time. Therefore, there are no preliminary results on these two groups' variances and means of these measures. This study's total sample size (*N* = 288) was determined to detect the effect size of 0.25 (Figure 4). For the same reason, the learning curve for neuromarker identification cannot be determined. However, one previous study determined that a test sample size of 100 would be needed to test a classifier to achieve reasonable precision in the validation (38).

## Discussion

To the best of our knowledge, this is the first large-scale (*N* = 288) multimodal MRI study of ME/CFS worldwide. While preliminary studies with a small sample size have shed some light on potential mechanisms of and factors associated with ME/CFS, a large scale study with well-established methodological principles is required for decisive conclusions (23), which is more critical for ME/CFS because of the controversy associated with this ill-defined disease (24). The recommendation for improving reproducibility, fully reporting analysis plans to reduce the file-drawer effect (23), has culminated in this protocol paper.

### Controlling confounding factors

ME/CFS is an illness currently defined by consensus criteria and questionnaires completed by patients. Thus, there is a risk of an ill-defined ME/CFS patient cohort. The proposed study will mitigate this problem by following CCC ME/CFS criteria and diagnosis agreed by two experienced clinicians (R.A.K and P.D.).

Patients with ME/CFS or fibromyalgia may have ongoing medications, which is logistically unavoidable for the study. We mitigate this risk by following steps. (1) Individuals using marijuana or substance were excluded because their known alteration of brain structure and function. (2) Each medication will be determined individually by agreement of investigators (Z.Y.S., R.A.K. and P.D.) on whether it affects the NVC. We include individuals on medication that does not affect the NVC. (3) Two clinicians (R.A.K. and P.D.) will assess individuals on medication affecting the NVC to determine if it is safe to stop medication for 3 weeks. Individuals considered safe to stop medication will further seek professional opinions from their caring physicians and then stop medication 2 weeks before and 1 week after MRI. We will exclude individuals who are at risk to stop medication affecting the NVC.

Both ME/CFS and fibromyalgia predominantly affect females (39). Thus, we may recruit more females than males. Indeed, our current sample includes 82% female and 18% male in all groups, including HCs. This unequal sex ratio risk cannot be avoided because of diseases' inherent features. We will include sex as a covariate in all statistical analyses.

This project started when the severe acute respiratory syndrome coronavirus 2 (SARS-CoV-2) occurred before the long Covid (post-acute sequelae of SARS-CoV-2 infection) was recognized. We decided to keep the study protocol and follow the CCC ME/CFS criteria while considering SAR-CoV-2 as an infection similar to other infections that occurred before the manifestation of ME/CFS.

### Disentangling NVC

The brain accounts for 20% of total body energy consumption but has limited or no energy reservoir (40). Thus, normal brain function relies critically on the timely matching of local blood flow to neural energy demand. If this matching is not achieved, then subtle brain changes ensue, culminating in chronic brain injury and associated cognitive impairment (6). The HRF indirectly measures NVC and is determined by neuronal activity, the brain vasculature, and Ca^2+^ signaling pathways. This study will combine HRF measurement with task-induced glutamate fluctuations (less affected by vasculatures) and breath-hold response functions (less affected by neuronal activities) to disentangle the NVC in ME/CFS.

Abnormal NVC has been observed in other brain disorders (5, 6). Promoting NVC function has already been explored from multiple aspects, including increasing global brain blood flow (41), modifying dietary (42), modulating calcium channels (43), and transcranial electrical stimulation (44). Therefore, the hypothesis of the proposed study, if confirmed, may lead to new evidence-based treatment design for ME/CFS.

### Neuromarker validation

Validation of a diagnosis marker should be capable of differentiating disease from HCs and, more importantly, differentiating disease from its comorbidity. Thus, this study will include groups of chronic fatigue and fibromyalgia. The chronic fatigue group in this study was defined as self-diagnosed or previously diagnosed CFS not confirmed by the two clinicians following CCC criteria. Fibromyalgia is the most frequent illness co-occurring with ME/CFS.

### Progress and plan

This study commenced in January 2020. The participant recruitment commenced in August 2020 and to date (20 May 2022), we have received and processed 902 expressions of interest and recruited 110 participants.

We will start preliminary analysis in July 2022 and disseminate preliminary results in October 2022. The final results and data release will be available in 2024.

### Ethics and dissemination

#### Ethics statement

This study was reviewed and approved by University of the Sunshine Coast Ethic committee with the approval number A191288.

This project was registered with The Australian New Zealand Clinical Trials Registry (ACTRN12622001095752).

### Results dissemination

The data from the study will be stored and archived by the UniSC library research data management plan. The full access of data will be limited to approved investigators only. Anonymised data will be transferred to password-protected computers in the USC computers for processing and analysis.

The disseminating results will not contain any personal information, and no participant will be able to be identified in any presentation or publication. Those results will be disseminated to researchers through scientific manuscripts and scientific conferences; to clinicians through clinician investigators; and to patients through social media such as Facebook and Twitter from our Institute and active engagement with ME/CFS associations.

The proposed neuromarker for diagnosis of ME/CFS requires MRI and differs from vital signs and blood tests. We acknowledge that its application into clinical practices may take a longer timeframe and extra effort. However, we also argue that many neurological disorders, such as vascular dementia (45) and multiple sclerosis (46), include MRI as an essential component. Indeed, MRI has become readily available, and 57 per 1,000 people in Australia have had an MRI scan between 2017 and 2021 (47).

## Ethics statement

The studies involving human participants were reviewed and approved by University of the Sunshine Coast University with the approval number A191288. The patients/participants provided their written informed consent to participate in this study.

## Author contributions

ZS conceived and designed the study. RK, PD, VC, SB, and JL participated in the study design. ZS, AM, TA, and SR developed the ethics protocol, participant recruitment, and data collection. RK and PD performed clinic interviews. All authors contributed to editing the manuscript.

## Funding

This project was supported by the National Health and Medical Research Council of Australia (NHMRC) Ideas Grant Scheme (GNT1184219) and The Mason Foundation (MAS2018F00024). VC was partly supported by NIH R01MH123610 and NSF 2112455.

## Conflict of interest

The authors declare that the research was conducted in the absence of any commercial or financial relationships that could be construed as a potential conflict of interest.

## Publisher's note

All claims expressed in this article are solely those of the authors and do not necessarily represent those of their affiliated organizations, or those of the publisher, the editors and the reviewers. Any product that may be evaluated in this article, or claim that may be made by its manufacturer, is not guaranteed or endorsed by the publisher.
